# Global Trends and Hotspots in Trigeminal Neuralgia Research From 2001 to 2021: A Bibliometric Analysis

**DOI:** 10.3389/fneur.2022.894006

**Published:** 2022-05-10

**Authors:** Ganggui Zhu, Zaixiang Fu, Sheng Su, Yajuan Tang, Fuyi Liu, Wenhua Yu

**Affiliations:** ^1^Department of Neurosurgery, Hangzhou First People's Hospital, School of Medicine, Zhejiang University, Hangzhou, China; ^2^Department of Neurosurgery, The Second Affiliated Hospital, School of Medicine, Zhejiang University, Hangzhou, China; ^3^Department of Neurosurgery, The Fourth Affiliated Hospital, School of Medicine, Zhejiang University, Yiwu, China

**Keywords:** bibliometric analysis, trigeminal neuralgia, CiteSpace, visualization, network analysis

## Abstract

**Background:**

In recent years, there have been an increasing number of studies on trigeminal neuralgia (TN). However, a scientific and comprehensive study of the current situation and trends in the field of TN research is lacking. The purpose of this study is to summarize and visualize the development, research hotspots, and future trends in TN based on a bibliometric approach.

**Methods:**

Studies on TN published from 2001 to 2021 were obtained from the Web of Science Core Collection (WoSCC). Bibliometrics, CiteSpace, and VOSviewer tools were used for bibliometric analysis and visualization.

**Results:**

In total, 4,112 documents were searched. The number of research articles in the field is generally on an upward trend, with the fastest growth in the number of articles from 2017 to 2020. Shanghai Jiao Tong University, Pittsburgh University, and Mayo Clinic are the three institutions with the most publications. Shiting Li and Zakrzewska JM are the most prolific author and top co-cited authors, respectively. *The Journal of Neurosurgery* is the most influential journal. The top 5 keywords in that time frame are TN, microvascular decompression, facial pain, stereotactic radiosurgery, and neuropathic pain.

**Conclusion:**

This is the first comprehensive scientific bibliometric analysis of the global research field on TN over the past 21 years, providing a meaningful reference for further exploration of topical issues and research trends in the field.

## Introduction

Classic trigeminal neuralgia (TN) is a disorder that is confined to the facial region and is characterized by brief, intense electric shock-like pain (1, 2). It is widely believed that compression of the trigeminal nerve near the brainstem by blood vessels causes demyelination of the sensory branches of the trigeminal nerve, resulting in TN (3, 4). There are multiple options regarding the treatment of TN, which also indicates that the treatment of TN is extremely challenging. Over the past 21 years, there has been an increasing interest in the nature, diagnostic strategies, and treatment of TN.

Dr. John Fothergill first publicly described TN in the 17th century (4). The usually preferred drug treatment for TN patients, Phenytoin, was the first drug approved for the treatment of TN (5). The European Federation of Neurological Societies (EFNS) guidelines suggest two first-line treatments for TN: carbamazepine and oxcarbazepine (6). However, due to reduced drug effectiveness and unavoidable side effects, microvascular decompression is becoming one of the most common surgical treatments for TN (7). Despite the increasing number of articles on TN, there has been a change in the focus of research due to the passage of time and the different research groups (8). As a result, it is often difficult for readers to quickly grasp the key and hot content in the TN field.

In-depth knowledge of historical and current research trends can provide valuable information about scientific advances and may assist in evidence-based clinical decision-making. Bibliometric analysis has become a powerful tool for the quantitative analysis of scholarly literature within a given field using a variety of methods (9, 10). According to us, there is no scientific quantitative analysis on TN study. To fill this gap, we performed a bibliometric analysis to quantitatively and qualitatively discuss TN studies published from 2001 to 2021. Specifically, the main objectives of this study were to provide an overview of the current status of the TN field, summarize major research clusters and popular research directions, and present prospects for the future development of the field.

## Materials and Methods

### Data Source

The bibliometric analysis is based on the Web of Science Core Collection (WOSCC), which is recognized as the most appropriate database for conducting bibliometric analysis (11). All data collection was performed on February 8, 2022, searching the WOSCC for all literature published between 2001 and 2021, using “trigeminal neuralgia” as the subject term. There is no restriction on the language used in the literature. Only articles of the original research and review type were included. A total of 4,112 articles were included in the study, and the exported literature was saved as all records and references were formatted and stored as plain text files in download_txt format.

### Data Analysis

Visual analysis uses Microsoft Excel 2019, Bibliometrix (R-Tool of R-Studio), VOSviewer, and CiteSpace.

The R tool Bibliometrix (Version 1.7) of R-Studio (Version 4.1.2) is used for comprehensive scientific cartographic analysis, combined with the web-based tool Biblioshiny to export and manage data from WoSCC. The basic data include aggregate information such as author name, corresponding author country, country/region, citations count, journal source, journal issue count, and keywords.

The VOSviewer (version 1.6.1) is a program for building and viewing bibliometric maps. It can be used to build author or journal maps based on collaborative data or to build keyword networks based on co-occurrence data.

Focused on analyzing the underlying knowledge contained in the scientific literature, CiteSpace is visual analysis software developed incrementally in the context of scientometrics and data visualization. The CiteSpace is used for authors, institutions, most frequently cited journals and references, and burst detection. It is also used for keyword burst detection and the timeline viewer.

Since all raw data used in this study were obtained from a public database, no ethical review was required.

## Results

### Overall Publication Performance

**Figure 1:**
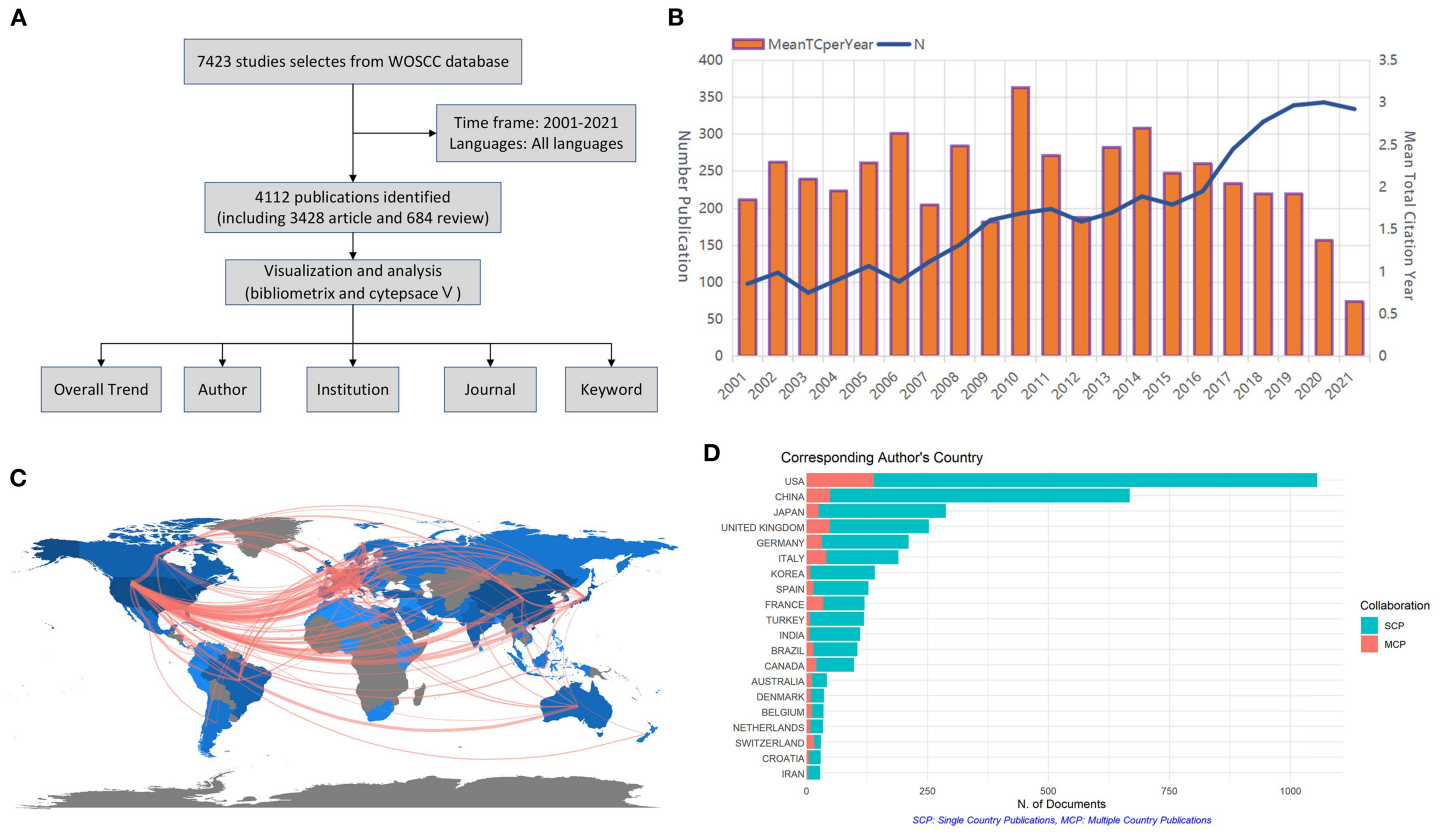
**(A)** Research flow chart. **(B)** General trends in publications 2001–2021. **(C)** World Collaborative Relationships Map. **(D)** Comparison of single-country publications and multiple country publications from different countries. SCP, single-country publications; MCP, multiple country publications.

The overall flow of the study is summarized in Figure 1A. The number of papers published in each period reflects the trend of research in this field. As shown in Figure 1B, the number of articles on TN showed an overall increasing trend. From 2001 to 2006, the annual output of publications in this period was approximately equal. From 2006 to 2016, the volume of literature steadily increased, and the average annual total citations of the literature reached its highest in 2010, indicating that TN is receiving increasing attention. From 2017 to 2020, the number of publications exploded, reaching 343 publication outputs in 2020. However, the volume of literature decreases in 2021.

### Countries and Regions

According to statistics, a total of 4,112 papers were published in 73 countries or regions during 2001–2021. The top ten most prolific countries and regions are given in Table 1. The country with the most publications is the United States (*n* = 1,205), followed by China (*n* = 610) and England (*n* = 230). Figure 1C shows the network diagram of cooperation between countries, which shows that there is strong cooperation between countries such as the United States, England, and China. Among the publications from different countries, the USA is in the first place in terms of the number of single country publications and multiple country publications, as shown in Figure 1D.

**Table 1:** Top 10 countries based on the total number of publications for the period 2001–2021.

**Rank**	**Country**	**Year**	**Centrality**	**Count (%)**
1	USA	2001	0.65	1,205 (23.90%)
2	China	2008	0.02	610 (14.83%)
3	Japan	2007	0.00	230 (5.59%)
4	England	2008	0.02	219 (5.33%)
5	Italy	2007	0.02	193 (4.69%)
6	Germany	2007	0.04	173 (4.21%)
7	South Korea	2007	0.00	145 (3.53%)
8	France	2007	0.07	121 (2.94%)
9	Canada	2008	0.00	107 (2.60%)
10	India	2008	0.00	106 (2.58%)

### Institutions

**Figure 2:**
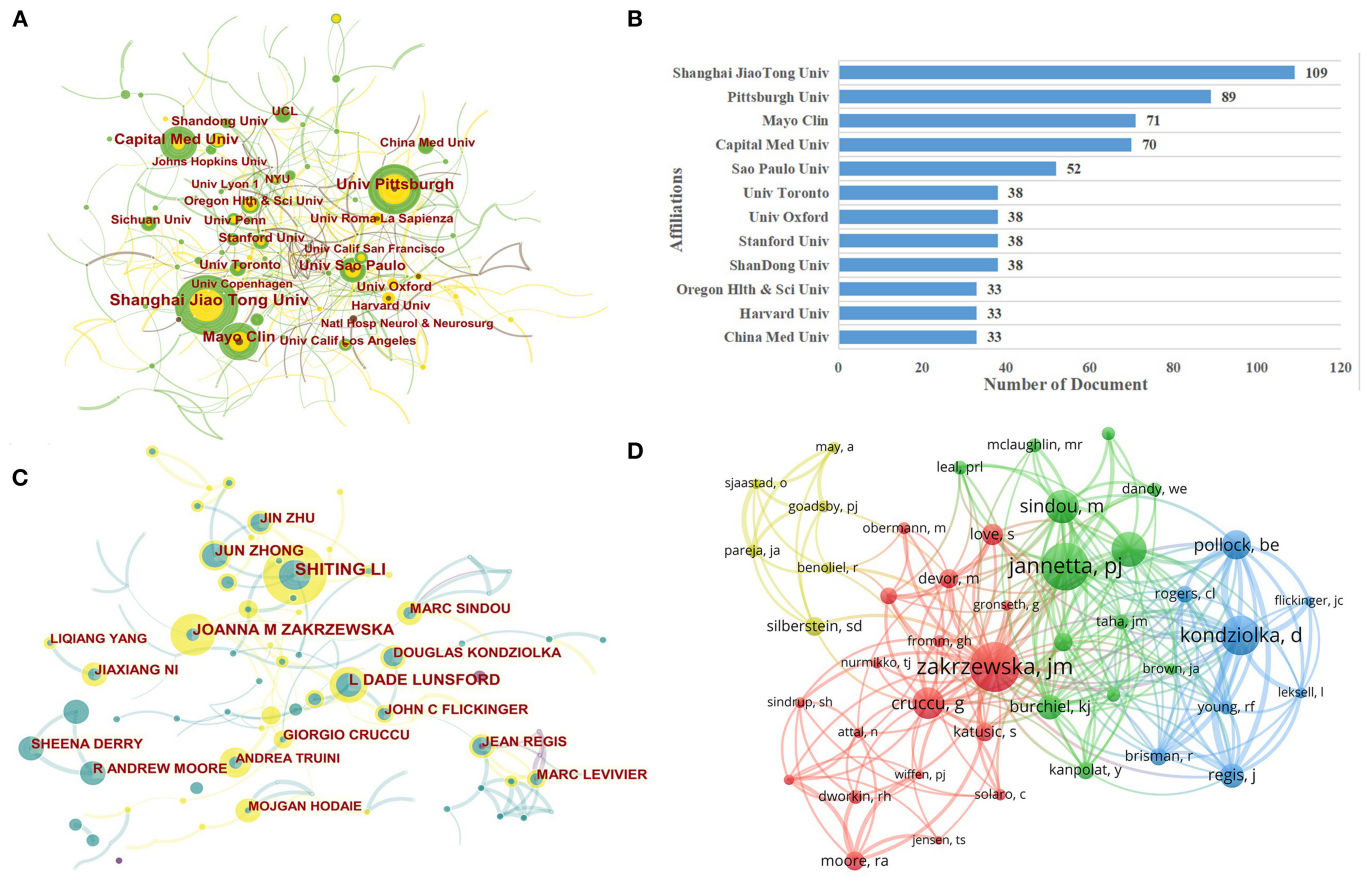
**(A)** The co-authorship network visualization map of institutions for trigeminal neuralgia (TN) research. **(B)** Top 12 most productive institutions between 2001 and 2021. **(C)** A CiteSpace visualization map of authors involved in TN. **(D)** A VOSviewer visualization map of co-cited authors involved in TN.

The cooperative relationship between the institutions is shown in Figure 2A. From this figure, it can be concluded that Shanghai Jiao Tong University, University of Pittsburgh, Mayo Clinic, and Capital Medical University are located in larger circles, representing the higher volume of publications from these institutions. The top ten most prolific institutions are shown in Figure 2B. Shanghai Jiao Tong University has the most publications (China, 109 publications), followed by Pittsburgh University (the USA, 89 publications) and Mayo Clinic (the USA, 71 publications).

### Authors and Co-Cited Authors

A total of 12,790 authors participated in the study of TN. The average number of authors per article was 3.1. Table 2 lists the top 10 authors involved in the study. Shiting Li was the most prolific author with 56 publications, closely followed by Zakrzewska JM (*n* = 37) and Lunsford LD (*n* = 36). CiteSpace visualizes the network between authors and collaborations, as shown in Figure 2C. Authors from the same country are well-connected and collaborate frequently, but the connections between different countries are still low.

**Table 2:** The top 10 prolific authors and co-cited authors on trigeminal neuralgia (TN) research from 2001 to 2021.

**Rank**	**Author**				**Co-cited authors**		
	**Name**	**Counts**	**Citations**	**Country**	**Name**	**Citations**	**Country**
1	ShiTing L	56	832	China	Zakrzewska JM	1,284	England
2	Zakrzewska JM	37	995	England	Jannetta PJ	1,221	USA
3	Lunsford LD	36	809	USA	Kondziolka D	1,000	USA
4	Zhong J	34	713	China	Barker, FG	871	USA
5	Kondziolka D	26	703	USA	Sindou M	829	France
6	Levivier M	26	529	Switzerland	Cruccu G	776	Italy
7	Regis J	26	742	France	Pollock BE	715	USA
8	Derry S	25	1,060	England	Burchiel KJ	589	USA
9	Moore R.A	25	1,060	England	Regis J	570	France
10	Cruccu G	24	1,754	Italy	Love S	500	England

The co-cited authors are authors who are cited by another paper or papers at the same time, and these authors constitute a co-citation relationship. The degree of citation is a key indicator of author contribution. The top 10 co-cited authors are shown in **Table 4**. Three authors were cited more than 1,000 times. The author Zakrzewska was the most frequently cited author with 1,284 citations. Figure 2 shows a network map based on VOSviewer with more than 150 co-cited authors. On the mapping, Zakrzewska JM is the most famous author.

### Journals and Co-Cited Academic Journals

We performed a visual analysis of the published literature using VOSviewer, which is summarized in Table 3. The *Journal of Neurosurgery* had the highest number of papers (*n* = 244, 5.93%), followed by *World Neurosurgery* (*n* = 216, 5.25%). Among the top 10 journals, the highest impact factor was the *Journal of Headache and Pain* (7.277 points). The higher the number of citations of a journal indicates the greater the impact of the journal. Three of the top ten cited co-cited journals have more than 5,000 citations. The *Journal of Neurosurgery* had the highest number of citations (*n* = 12,225), followed by *Neurosurgery* (*n* = 8,924). According to the 2020 Journal citation reports (JCR), 80% of the top ten journals are located in the Q2 and above region.

**Table 3:** Top 10 journals and co-cited journals related to TN.

**Rank**	**Journal**					**Co-cited Journal**			
	**Name**	**Count**	**Citation**	**IF**	**JCR**	**Name**	**Co-citation**	**IF**	**JCR**
1	Journal of neurosurgery	244 (5.93%)	6,771	5.115	Q2	Journal of Neurosurgery	12,225	6.771	Q2
2	World neurosurgery	216 (5.25%)	1,515	2.104	Q4	Neurosurgery	8,924	4.654	Q2
3	Neurosurgery	150 (3.64%)	5,283	4.654	Q2	Pain	6,414	6.961	Q2
4	Acta neurochirurgica	139 (3.38%)	2,637	2.216	Q4	Neurology	4,810	9.91	Q1
5	Journal of clinical neuroscience	76 (1.85%)	1,083	1.961	Q4	Cephalalgia	4,299	6.292	Q2
6	Cephalalgia	75 (1.82%)	1,797	6.292	Q2	Acta Neurochirurgica	3,300	2.216	Q4
7	Headache	75 (1.82%)	1,251	5.887	Q2	Headache	2,534	5.887	Q2
8	Clinical neurology and neurosurgery	70 (1.7%)	877	1.876	Q4	Journal of Neurology Neurosurgery and Psychiatry	1,849	10.15	Q1
9	Journal of craniofacial surgery	64 (1.56%)	438	1.046	Q4	Stereotactic and Functional Neurosurgery	1,635	1.875	Q4
10	Journal of headache and pain	64 (1.56%)	1,265	7.277	Q2	Brain	1,555	13.5	Q1

**Figure 3:**
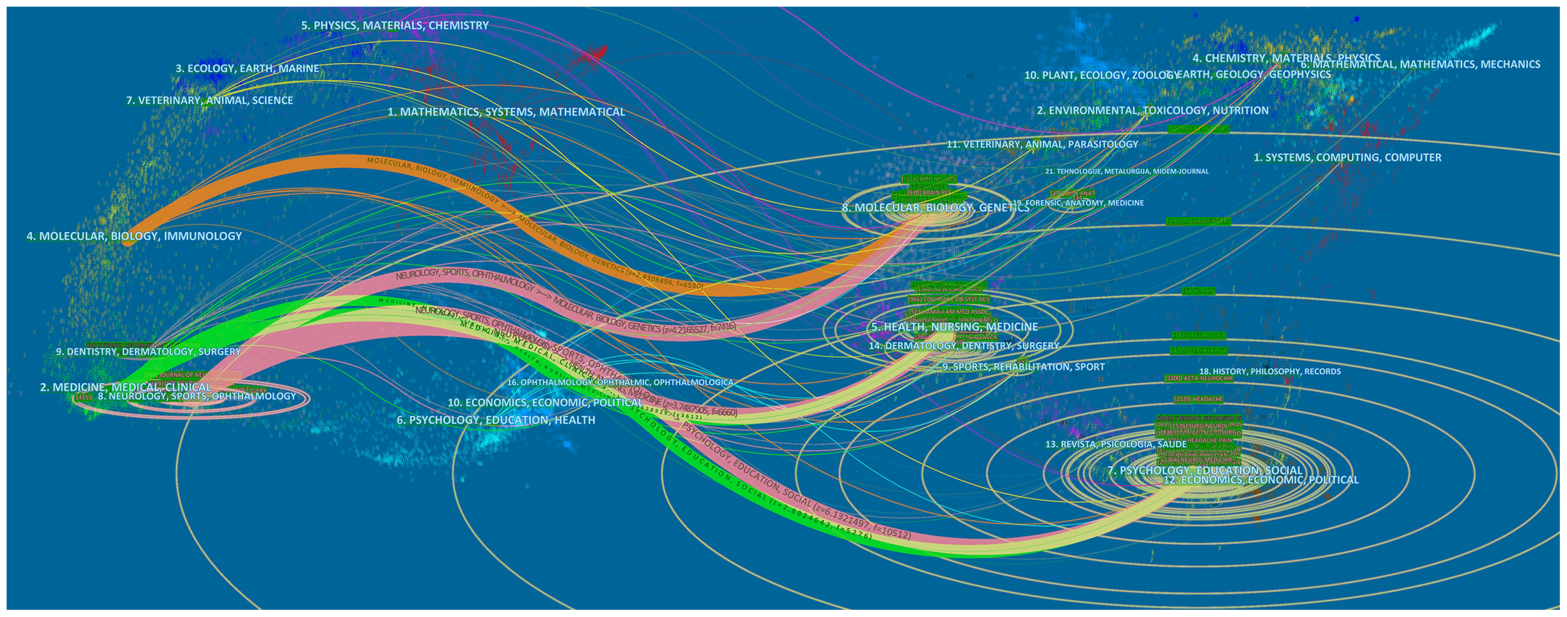
The dural map overlay of journals on TN.

The double map of journals shows the citing journals on the left and the cited journals on the right, with the colored paths between them indicating the citation relationship. The orange path in Figure 3 indicates that the literature published in molecular/biology/genetics journals is frequently cited in Molecular/Biology/Immunology and Neurology/Sports/ophthalmology journals.

### Co-Cited References and References

**Figure 4:**
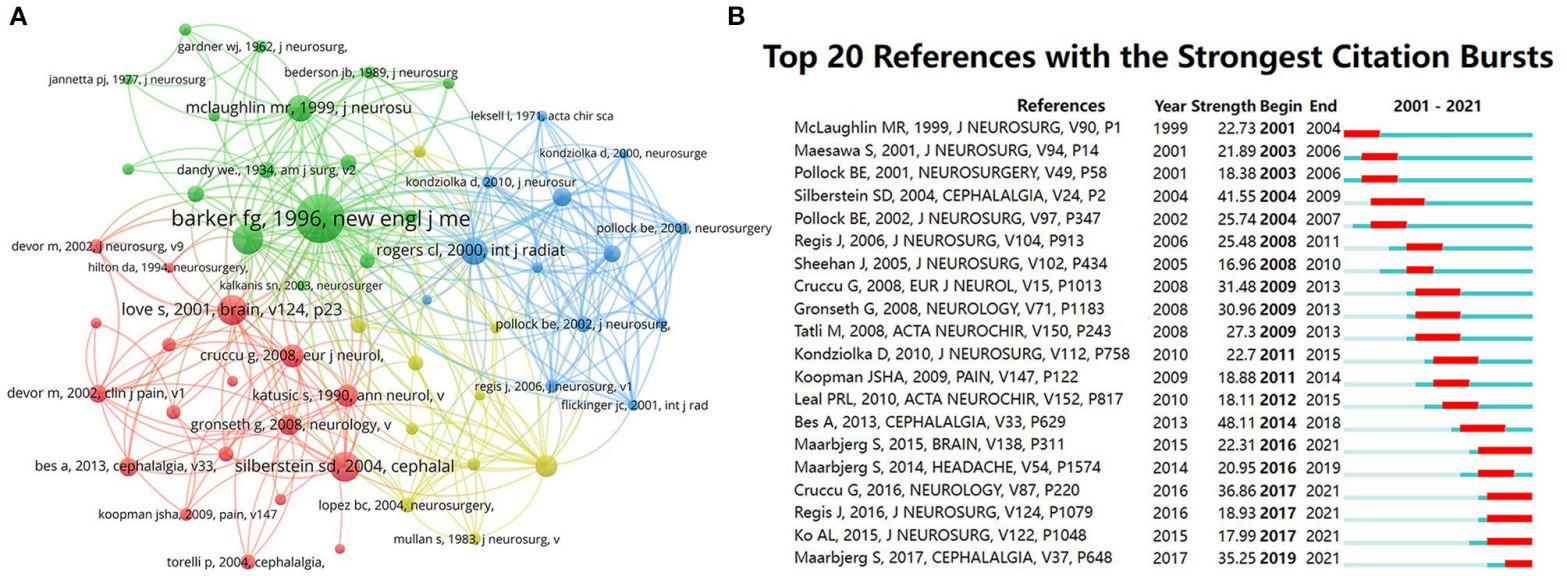
**(A)** The co-citation network visualization map of references on TN research between 2001 and 2021. **(B)** The top 20 references with the strongest citation bursts on TN research from 2001 to 2021. The red segment of the blue line denoted the burst duration of a keyword.

A co-cited reference is when two or more articles appear in the reference list of other literature at the same time, which reflects the citation relationship between the works of literature. Out of 4,112 papers, 65,153 co-cited references were identified. The 10 most cited papers are shown in Table 4. *The long-term outcome of microvascular decompression for trigeminal neuralgia* had the highest number of citations (*n* = 625) (12). Among the networks co-cited in the literature in Figure 4A, the study was the most influential, with strong relationships to the rest of the literature.

**Table 4:** Top 10 most cited references.

**Rank**	**Cited reference**	**Citation**	**Year**	**Journal**	**First author**
1	The long-term outcome of microvascular decompression for trigeminal neuralgia	625	1996	The New England Journal of Medicine	Fred G. Barker
2	Structural mechanisms of trigeminal neuralgia	361	1967	Journal of Neurosurgery	Peter J. Jannetta
3	Migraine pathophysiology and its clinical implications	345	2004	Cephalalgia	Stephen D. Silberstein
4	Trigeminal neuralgia: pathology and pathogenesis	343	2001	Brain	Seth Love
5	Gamma knife radiosurgery for trigeminal neuralgia: the initial experience of The Barrow Neurological Institute	314	2000	International Journal of Radiation Oncology Biology Physics	C. Leland Rogers
6	Microvascular decompression of cranial nerves: lessons learned after 4,400 operations	296	1999	Journal of Neurosurgery	Mark R. Mclaughlin
7	AAN-EFNS guidelines on trigeminal neuralgia management	253	2008	European Journal of Neurology	Cruccu Giorgio
8	Percutaneous controlled radiofrequency trigeminal rhizotomy for the treatment of idiopathic trigeminal neuralgia: 25-year experience with 1,600 patients	246	2001	Neurosurgery	Yucel Kanpolat
9	Incidence and Clinical Features of Trigeminal Neuralgia, Rochester, Minnesota, 1945–1984	240	1990	Annals of Neurology	Slavica Katusic
10	Practice Parameter: The diagnostic evaluation and treatment of trigeminal neuralgia (an evidence-based review)	226	2008	Neurology	Gary S. Gronseth

Citation bursts reflect the references of interest to researchers at a given time. The CiteSpace V was used to identify the references with the strongest citation bursts. Figure 4B shows that the burst values of the top 20 documents with the strongest citation bursts fluctuate between 16.96 and 48.11. “Bes A, 2013, CEPHALALGIA, V33, P629” has the strongest citation burst (48.11) (13). “Maarbjerg et al., BRAIN, V138, P311” (14) had the longest burst duration of 5 years. A total of five co-citations had the most recent burst.

### Keywords and Hotspots

**Figure 5:**
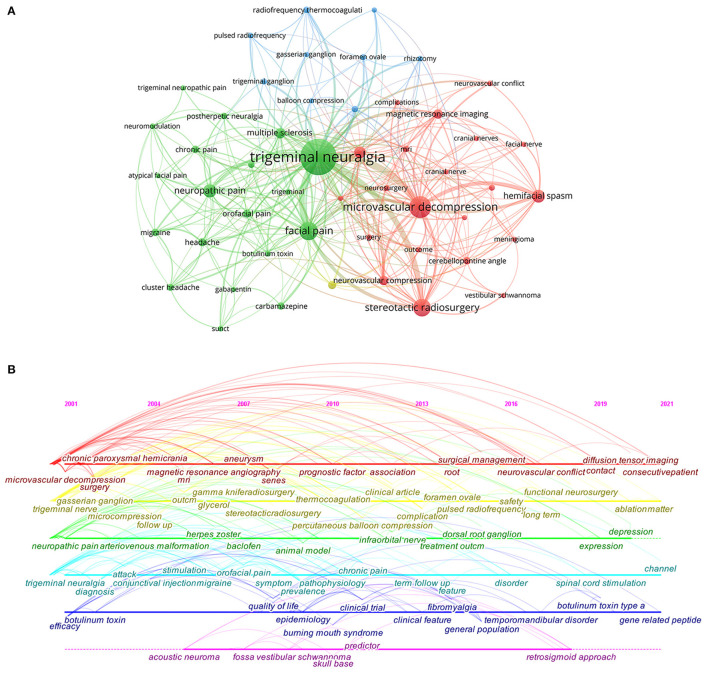
**(A)** The co-citation network visualization map of keywords on TN research between 2001 and 2021. **(B)** A CiteSpace visualization map of timeline viewer related to TN.

A total of 5,894 keywords were extracted from 4,112 documents. Limiting the number of occurrences to 30 or more, 50 keywords were identified. The top five keywords were TN (*n* = 1,958), microvascular decompression (*n* = 675), facial pain (*n* = 465), stereotactic radiosurgery (*n* = 418), and neuropathic pain (*n* = 227). Figure 5 shows the co-occurrence network of keywords. The keywords are divided into 4 clusters. Cluster 1 (red) deals with surgical treatment and diagnostic imaging, such as microvascular decompression, cerebellopontine angle, complications, diffusion tensor imaging, and magnetic resonance imaging. Cluster 2 (green) has keywords mainly related to clinical manifestations and comorbidities, such as TN, cluster headache, multiple sclerosis, and postherpetic pain. Cluster 2 (green) is related to minimally invasive interventions such as percutaneous puncture balloon compression, trigeminal ganglion, and radiofrequency thermocoagulation (RFT).

The TN timeline viewer drawn based on CiteSpace software visualizes the phase hotspots and development directions of TN research from the time dimension. In the first decade (2001–2010), the study focused on the exploration of the treatment and mechanism of TN, with keywords such as TN, microvascular decompression, stereotactic radiotherapy, and animal models. The study during 2011–2021 focused on the management and prognosis of TN after treatment, with the main keywords: clinical feature, treatment outcome, term follow-up, safety, and diffusion tensor imaging.

Keyword bursts can be used to reflect hotspots, frontiers, and trends in the research field (15). “Safety” is the strongest emergent strength (14.87) and continues to emerge as a keyword until 2021. In addition, the keywords with the most recent bursts include “pulsed radiofrequency,” “vestibular schwannoma,” “efficacy,” and “thermocoagulation.”

## Discussion

In this study, we present the overall research results of TN over the past 21 years. The number of papers and trends in the literature each year may reflect the rate of development and research progress of the study. From 2001 to 2006, the number of papers related to TN each year was approximately equal, and research on TN is still in its early stages. From 2006 to 2016, the number of publications increased steadily, which may reflect the clinical heterogeneity and the diversity of TN research areas. After 2017, the number of TN studies increased significantly, reflecting the concentration of research in the areas of diagnosis, treatment, and international standards of TN.

According to Table 1, the country with the highest number of literature publications is the USA (*n* = 1205, 23.90%), followed by China (*n* = 610, 14.83%), and Japan (*n* = 230, 5.59%), which together account for about 40% of the total. This indicates that the United States, China, and Japan are most concerned with the development of the TN domain. Centrality is a measure of the importance of nodes in the network graph (16). Among the countries with the highest top 10 publications (Table 1), the USA has the highest centrality (0.65), which implies that the USA dominates the global TN research field. As a developed country, the USA owns numerous research institutions and invests a lot of money in research (17–19). Among the top 12 research institutions, five are situated in the United States and four are in China. However, as seen in Figure 2A, the location relationship among institutions is relatively scattered, indicating a lack of academic cooperation and communication among institutions. Therefore, each country and each institution should continue to strengthen communication and cooperation to jointly promote the research and development of TN.

Analysis of literature sources can help researchers to find the core journals in their field of study, and publications from the top co-cited journals can be used as authoritative references (20). As shown in Table 3, the *Journal of Neurosurgery* published the most articles while having the most co-cited articles. In addition, three journals were in the top 10 of both prolific and co-cited journals: *Journal of Neurosurgery, Neurosurgery*, and *Pain*. Most of the co-cited journals had impact factors of 5 or more, and two journals originated from Q1: *Neurology* and *Journal of Neurology Neurosurgery and Psychiatry*. These co-cited journals are highly recommended by researchers in the field. The most cited article was the retrospective analysis of the long-term outcomes after microvascular decompression for TN by Barker et al. (12). Another clinical study related to microvascular decompression was written by Mclaughlinet et al. (21). Of the ten most cited articles, most types of literature are of the review type, focusing on the pathology, pathogenesis, diagnosis, and treatment of TN. These studies provide new insights into the management of TN.

**Figure 6:**
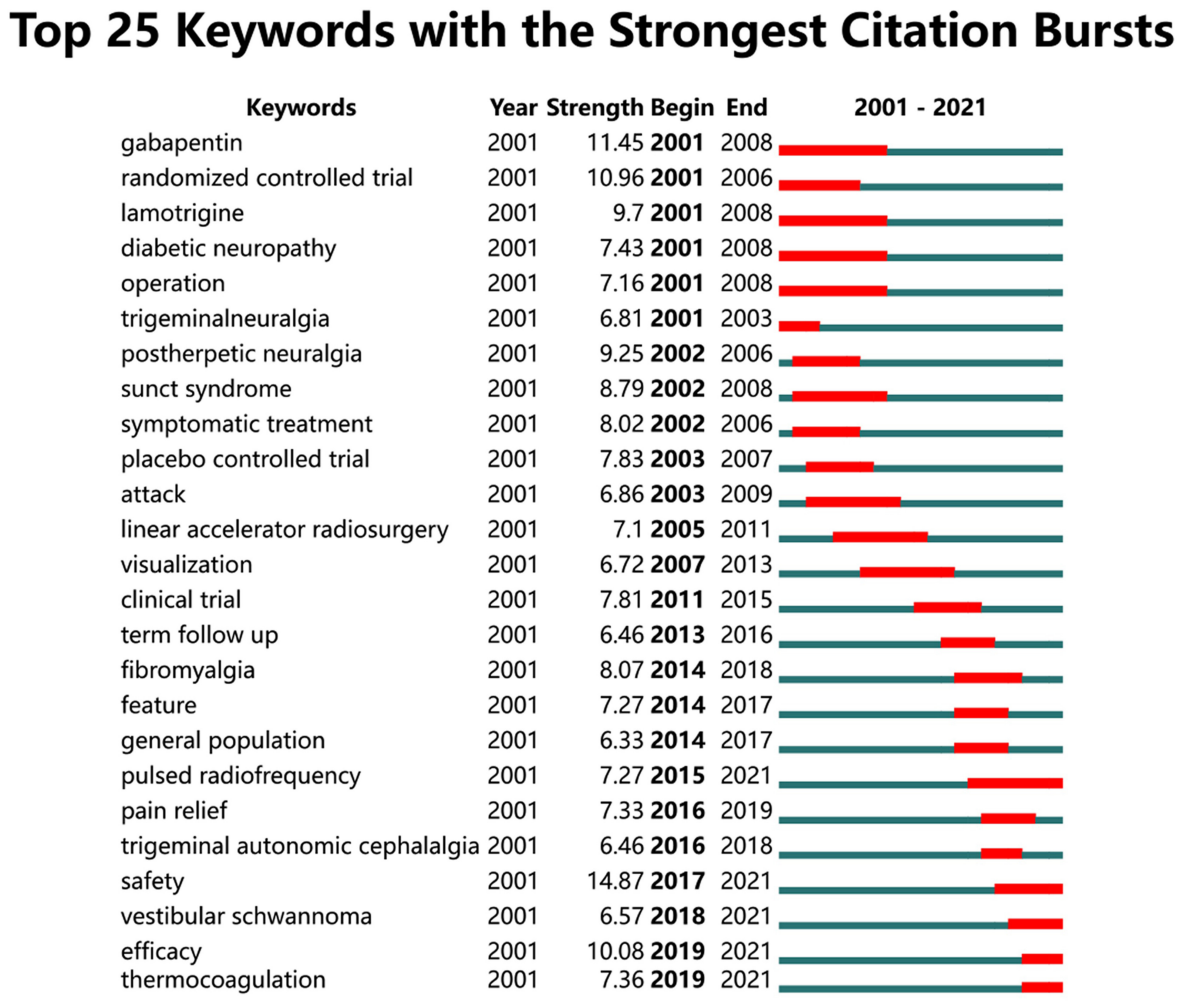
The top 25 keywords with the strongest citation bursts on TN research from 2001 to 2021.

The keywords' co-occurrence network and bursts reflect the research hotspots and development dynamics in the field of TN (22). As shown in Figure 5, microvascular decompression is most prominent in keyword cluster 1 (red), which represents the international attention and recognition of this treatment modality. As early as the 1950s, Gardner and Miklos et al. proposed the most classic intervention in the treatment of TN to date: microvascular decompression (23). Microvascular decompression (MVD) is recommended for patients who have failed drug therapy, experienced severe side effects, or relapsed (24, 25). In general, the prognostic of MVD is satisfactory. Results of large numbers of cases have shown that the immediate pain relief rate after microvascular decompression exceeds 90%, and 80% of patients remain pain-free at 1-year follow-up (26–28). However, in some cases, MVD is not the best choice. For the elderly who cannot tolerate the perioperative risks, percutaneous surgery seems to be a better option. As shown in Figure 6, pulsed radiofrequency (PRF) and RFT have been gaining a high level of attention in recent years. The main principle of radiofrequency (RF) therapy is to destroy the nerve at high temperature to block the pain signal transmission (RFT) or to modulate the nerve function of the trigeminal nerve at low temperature (PRF) (29). The RF treatment is similarly effective to MVD (30). The control of temperature during RF remains a key concern; researchers believe that RFT at 68–75°C results in better relief rates and fewer complications (31, 32). In addition, it has been suggested that PRF combined with RFT may improve clinical outcomes (29). However, more clinical data are still needed to validate this idea. Stereotactic radiosurgery is the least invasive procedure and a commonly accepted treatment for refractory TN (33). However, the short duration of pain relief is one of the reasons why it has not been chosen by many neurosurgeries. As the researchers are concerned, pain relief or efficacy is the focus of TN management. However, more importantly, safety is something that researchers have always cared about regarding the treatment of TN, especially in the field of surgical treatment. Although the risk of serious complications from MVD surgery is <2%, the overall surgical complication rate is as high as 20% (27, 28, 34, 35). Common complications include trigeminal numbness and dullness of sensation, hearing loss, facial nerve palsy, cerebrospinal fluid leakage, etc. (34). Therefore, it is still the direction of scholars' efforts to reduce surgical complications and improve efficacy.

Although the understanding of TN has gradually deepened, there are still many directions that deserve attention. Further exploration of the etiology and pathophysiology of TN may bring more effective treatment. Idiopathic TN is more common in female patients, and the age of onset is usually younger (36, 37). However, the mechanisms responsible for this difference remain unknown. Therefore, research on gender-related factors in TN may provide the basis for future individualized treatment. The development of magnetic resonance imaging, in particular, diffusion tensor imaging, provides abnormal neural structures that cannot be detected by conventional means. A study from Canada suggests that microstructural diffusivities of the trigeminal nerve in the pontine segment of the brain can be used to predict surgical remission rates (38). Moreover, using brain gray matter analysis, Michael et al. found significant reductions in cortical and subcortical thickness volumes in areas regulating pain in patients with TN, including specific hippocampal subregion volumes (39). The application of neuroimaging may provide new perspectives for exploring the etiology of TN. Surgery is the option for most patients with TN after pharmacological treatment has failed. However, the appropriate timing of surgical intervention remains to be determined. For both views, early surgery for non-response to the first-line drug therapy vs. at least two drug treatments followed by surgery have its advantages and drawbacks (40). Furthermore, as a common treatment for refractory TN with arterial neurovascular compression, little is known about the outcome after MVD for TN with venous compression alone. A meta-analysis showed that patients with venous compression alone had a similar rate of pain relief after MVD compared to patients with arterial compression, but had a higher recurrence rate (41). Therefore, further studies are needed for the treatment of patients with venous compression.

There are still some limitations in this study. First, due to the limitation of CiteSpace software, we only extracted articles from the Web of Science database, and the literature that failed to be included in WoSCC was not included in the analysis, which may lead to publication bias. Second, this study was extracted from 2001 to 2021. With the efforts of researchers and the continuous updating of the literature, the findings of this study may be different from the realistic results.

## Conclusion

To our knowledge, this review is the first bibliometric study to scientifically and systematically analyze the global TN research trends over the past 21 years. This study can help scholars to understand the hotspots and frontiers in TN research and guide them to choose new research directions. Further cooperation among authors, institutions, and countries in the future is expected to accelerate the development of TN research and explore more effective management tools.

## Data Availability Statement

The original contributions presented in the study are included in the article/supplementary material, further inquiries can be directed to the corresponding author/s.

## Author Contributions

GZ was responsible for the conceptualization of the study, the design of the idea, and the writing of the first draft. GZ, ZF, and SS were responsible for collecting data and visualizing the results. YT and FL validated the visualization results. WY provided guidance throughout the work and proofed the manuscript. All authors agreed to approved the final manuscript.

## Funding

This study was financially supported by the Department of Neurosurgery, Hangzhou First People's Hospital, School of Medicine, Zhejiang University.

## Conflict of Interest

The authors declare that the research was conducted in the absence of any commercial or financial relationships that could be construed as a potential conflict of interest.

## Publisher's Note

All claims expressed in this article are solely those of the authors and do not necessarily represent those of their affiliated organizations, or those of the publisher, the editors and the reviewers. Any product that may be evaluated in this article, or claim that may be made by its manufacturer, is not guaranteed or endorsed by the publisher.
